# Polymorphonuclear myeloid-derived suppressor cells impair the anti-tumor efficacy of GD2.CAR T-cells in patients with neuroblastoma

**DOI:** 10.1186/s13045-021-01193-0

**Published:** 2021-11-12

**Authors:** Nicola Tumino, Gerrit Weber, Francesca Besi, Francesca Del Bufalo, Valentina Bertaina, Paola Paci, Linda Quatrini, Laura Antonucci, Matilde Sinibaldi, Concetta Quintarelli, Enrico Maggi, Biagio De Angelis, Franco Locatelli, Lorenzo Moretta, Paola Vacca, Ignazio Caruana

**Affiliations:** 1 Immunology Research Area, IRCCS Bambino Gesù Children’s Hospital, Viale San Paolo 15, 00146 Rome, Italy; 2 Department of Pediatric Hematology and Oncology, Cell and Gene Therapy, IRCCS Bambino Gesù Children’s Hospital, Piazza Sant’Onofrio, 4, 00165 Rome, Italy; 3 Department of Pediatric Hematology, Oncology and Stem Cell Transplantation University Children’s Hospital of Würzburg, 97080 Würzburg, Germany; 4 Institute for Systems Analysis and Computer Science “Antonio Ruberti”, National Research Council, Rome, Italy; 5 Department of Maternal, Infantile, and Urological Sciences, Sapienza University of Rome, Rome, Italy

**Keywords:** Neuroblastoma, Polymorphonuclear myeloid-derived suppressor cells, GD2.CAR T-cells, Clinical response, T-cell functionality, Long-term response

## Abstract

The outcome of patients affected by high-risk or metastatic neuroblastoma (NB) remains grim, with ≥ 50% of the children experiencing relapse or progression of the disease despite multimodal, intensive treatment. In order to identify new strategies to improve the overall survival and the quality of life of these children, we recently developed and optimized a third-generation GD2-specific chimeric antigen receptor (CAR) construct, which is currently under evaluation in our Institution in a phase I/II clinical trial (NCT03373097) enrolling patients with relapsed/refractory NB. We observed that our CAR T-cells are able to induce marked tumor reduction and even achieve complete remission with a higher efficiency than that of other CAR T-cells reported in previous studies. However, often responses are not sustained and relapses occur. Here, we demonstrate for the first time a mechanism of resistance to GD2.CAR T-cell treatment, showing how polymorphonuclear myeloid-derived suppressor cells (PMN-MDSC) increase in the peripheral blood (PB) of NB patients after GD2.CAR T-cell treatment in case of relapse and loss of response. In vitro, isolated PMN-MDSC demonstrate to inhibit the anti-tumor cytotoxicity of different generations of GD2.CAR T-cells. Gene-expression profiling of GD2.CAR T-cells “conditioned” with PMN-MDSC shows downregulation of genes involved in cell activation, signal transduction, inflammation and cytokine/chemokine secretion. Analysis of NB gene-expression dataset confirms a correlation between expression of these genes and patient outcome. Moreover, in patients treated with GD2.CAR T-cells, the frequency of circulating PMN-MDSC inversely correlates with the levels of GD2.CAR T-cells, resulting more elevated in patients who did not respond or lost response to the treatment. The presence and the frequency of PMN-MDSC in PB of high-risk and metastatic NB represents a useful prognostic marker to predict the response to GD2.CAR T-cells and other adoptive immunotherapy. This study underlines the importance of further optimization of both CAR T-cells and clinical trial in order to target elements of the tumor microenvironment.

**Supplementary Information:**

The online version contains supplementary material available at 10.1186/s13045-021-01193-0.

Chimeric antigen receptor (CAR) T-cell technology has rapidly evolved during the past decade, offering unprecedented results and now commercially available CAR T-cells targeting CD19 are employed to treat patients with B-cell malignancies non-responding to conventional therapy [1]. On the contrary, a big challenge remains the treatment of refractory solid tumors with CAR T-cells. Indeed, while pre-clinical studies showed encouraging results, only partial and transient responses have been obtained in clinical trials [2, 3]. These studies, however, uncovered mechanisms which may explain the limited success of CAR T-cell therapy, including lack of CAR T-cell persistence, quality of targeted antigen and the suppressive effect of the tumor microenvironment (TME) [2, 4]. Several strategies to overcome these mechanisms have been explored, including the targeting of regulatory/suppressive immune cells responsible of the TME-mediated inhibition [5–8]. Myeloid-derived suppressor cells (MDSC) are immature myeloid cells that arise from bone-marrow (BM) myeloid progenitors. Within the MDSC population, two main subsets can be identified: monocytic (CD45^+^, Lin^−^, HLA-DR^−/low^, CD33^+^, CD11b^+^, CD14^+^, CD66b^−^ and CD15^−^) and polymorphonuclear (PMN) (CD45^+^, Lin^−^, HLA-DR^−/low^, CD33^+^, CD11b^+^, CD14^−^, CD66b^+^ and CD15^+^) cells [9]. The expansion of these myeloid cell subsets has been detected in several conditions characterized by a high level of inflammation, including cancer [10], autoimmune disorders [11, 12], sepsis and infectious diseases [13, 14]. MDSC are recruited by solid tumors and consist of immature cells contributing to the establishment of an immunosuppressive microenvironment [15].

MDSC presence has been documented in patients with different tumors and their potent immunosuppressive activity is now widely recognized [16]. Recently, in a mouse model, the expansion of MDSC has been reported to decrease CAR T-cell efficacy in metastatic liver tumors [17] and interventions to inhibit MDSC showed anti-tumor benefit in a murine sarcoma model [5].

In this study, we show that PMN-MDSC with inhibitory activity are highly enriched in peripheral blood (PB) of Neuroblastoma (NB) patients with refractory/relapsed disease after treatment of GD2.CAR-T cells (Fig. 1A, B, Additional file 1: Fig. S1). These patients, in whom multi-modal conventional treatments had failed, were enrolled at our Institution in a clinical trial exploring the safety/efficacy of a novel GD2.CAR T-cell product [18] (Additional file 6: Supplementary materials and methods). First, we investigated whether PMN-MDSC could be involved in GD2.CAR T-cell functional impairment. To this end, we showed that allogeneic PMN-MDSC, obtained from hematopoietic stem-cell transplantation-donors undergoing mobilization with G-CSF [9], display a marked inhibitory effect on several second- and third-generation GD2.CAR T-cells (Fig. 1C, D, Additional file 2: Fig. S2, Additional file 3: Fig. S3).

**Fig. 1:**
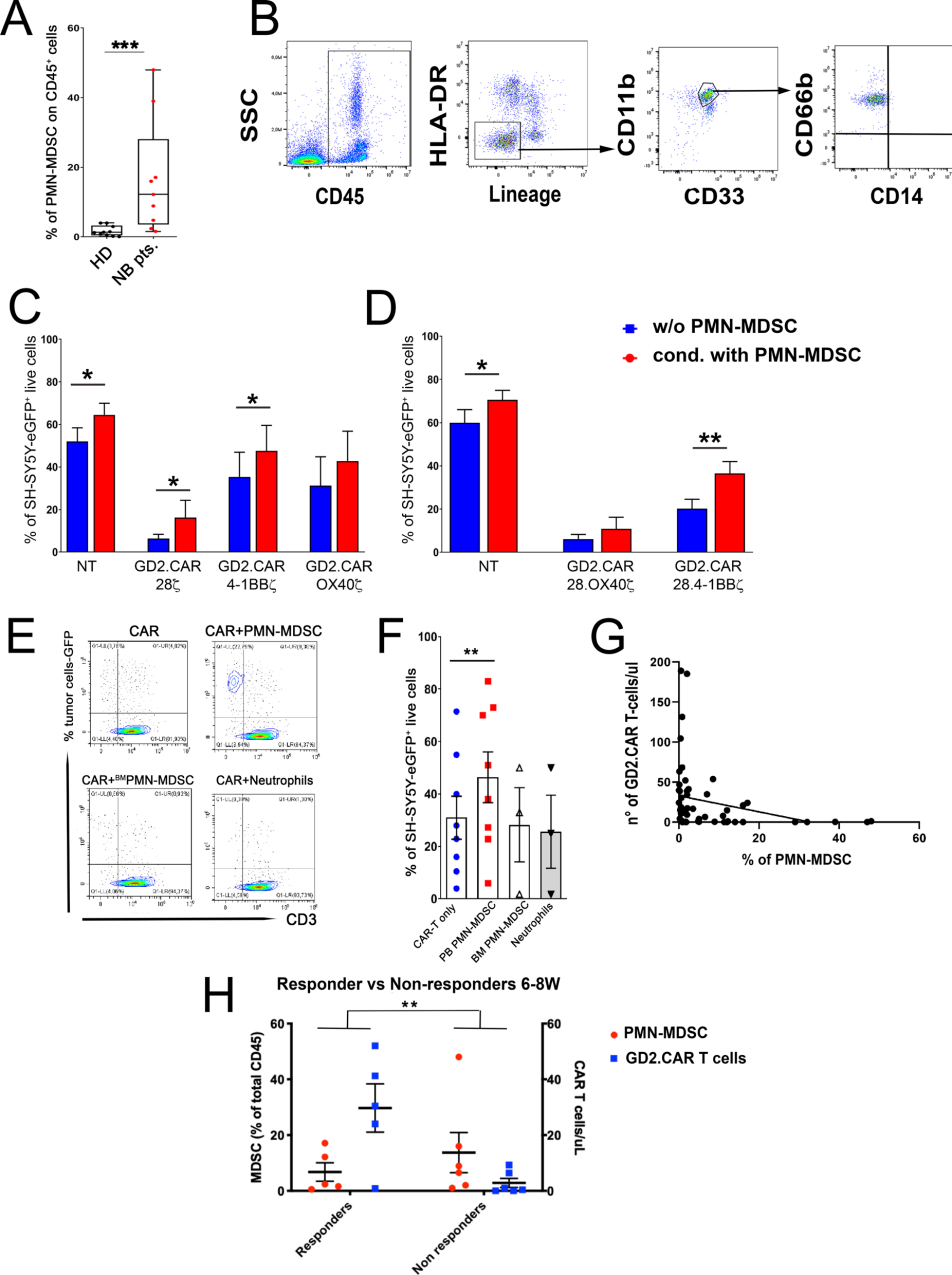
Presence of PMN-MDSC in PB of NB patients and their effect on GD2.CAR T-cells. **A**, **B** Mononuclear cells isolated from the PB of NB patients were analyzed by flow-cytometry for the expression of specific markers allowing the identification of PMN-MDSC subsets. **A** Percentages of PMN-MDSC on CD45^+^cells (CD66b^+^CD14^−^cells) in healthy donors (HD, *n* = 10) and in NB patients (NB pts, *n* = 9). **B** A representative gating strategy for PMN-MDSC identification by flow-cytometry is shown. **C**, **D** Second- and third-generation GD2.CAR T-cells were cultured either in the absence (w/o, blue bars) or in the presence (cond, red bars) of PMN-MDSC collected from stem cell donors given G-CSF and undergoing to leukapheresis. After 48h, GD2.CAR T-cells and non-transduced (NT) cells (used as control) were collected, purified and co-cultured at the effector:target ratio 1:1 with the SH-SY5Y-eGFP^+^ NB cell line. Percentages of SH-SY5Y-eGFP^+^ NB residual live cells at day 5 of co-culture with (**C**) second- (*n* = 10) and **D** third- (*n* = 10) generation GD2.CAR T-cells are reported. Patient derived GD2.CAR T-cells expressing CD28.4-1BBζ (the same used in our clinical trial) were cultured in the presence of PB-derived (*n* = 8) or BM-derived (*n* = 3) PMN-MDSC or PB-derived neutrophils (*n* = 3) collected from NB patients. After 48h, GD2.CAR T-cells were collected, purified from PMN-MDSC/neutrophils as described in supplementary data (Additional file 6), and co-cultured at an Effector:Target (E/T) ratio 1:1 with SH-SY5Y-eGFP^+^ NB cell line for 3 days. Percentages of SH-SY5Y-eGFP^+^ NB residual live cells were analyzed. **E** One representative co-culture experiment of GD2.CAR T-cells and PMN-MDSC is shown. **F** Percentages of residual live NB tumor cells in different culture conditions is reported. **G** Correlation between PMN-MDSC and GD2.CAR T-cells in CD45^+^ cells in NB patients (*n* = 55, different time point after GD2.CAR T-cell infusion), *r*^2^ = 0.07 and *p* value is indicated. Mononuclear cells present in the PB of NB patients were analyzed by flow-cytometry. Graphs represent percentages of PMN-MDSC and the corresponding absolute number/µl of GD2.CAR T-cells in NB patients. **H** Graphs represent GD2.CAR T-cells/µl (blue squares) and the percentages of PMN-MDSC (red dots) in CD45^+^ cells in responder (*n* = 5) and non-responder (*n* = 6) patients (multiple comparison with mixed-effect analysis). *p < 0.05, **p < 0.01, ***p < 0.005. Where not indicated, data were not statistically significant. **A** Mann–Whitney and **C**, **D**, **F** Wilcoxon Student’s *t* test. Data are shown as mean ± standard error of the mean (SEM)

To corroborate our data, we analyzed the inhibitory activity of PMN-MDSC that were collected from PB of GD2.CAR T-cell-treated-NB-patients. Our results confirmed the strong inhibitory capability of these cells on third-generation GD2.CAR T-cells in vitro, while neither autologous PB neutrophils nor BM low-density neutrophils, inhibit GD2.CAR T-cell cytotoxicity (Fig. 1E, F, Additional file 1: Fig. S1).

PMN-MDSC could be detected after GD2.CAR T-cell treatment and, importantly, their frequency in PB inversely correlated with that of GD2.CAR T-cells (Fig. 1G). Although, the number of patients analyzed in the present study is limited, we could observe that in patients with an early expansion of GD2.CAR T-cells a very low percentage of PMN-MDSC was observed. Of note, these patients (Responder) were characterized by a good response to therapy, reflecting a potent GD2.CAR T-cell activation. In non-responder patients, as well as in patients that lost response, high percentages of PMN-MDSC were associated with a lack of substantial expansion of GD2.CAR T-cells (Fig. 1H, Additional file 4: Fig. S4).

To better define the molecular mechanisms of the inhibitory effect, we performed a gene-expression profiling on PMN-MDSC-conditioned GD2.CAR T-cells. We observed modulation of numerous genes (*p* ≤ 0.01) involved in inflammation, cell activation, signal transduction and extra-cellular communication (Fig. 2A–C, Additional file 5: Fig. S5A), as well as cytokine/chemokine secretion (Fig. 2D). Three genes were found to be significantly upregulated in all conditioned T-cells: S100A8, S100A9 and TNFAIP6. These genes are involved in the immune response, leukocyte activation, inhibitory pathways induced by cell surface receptors, extracellular communications and others.

**Fig. 2:**
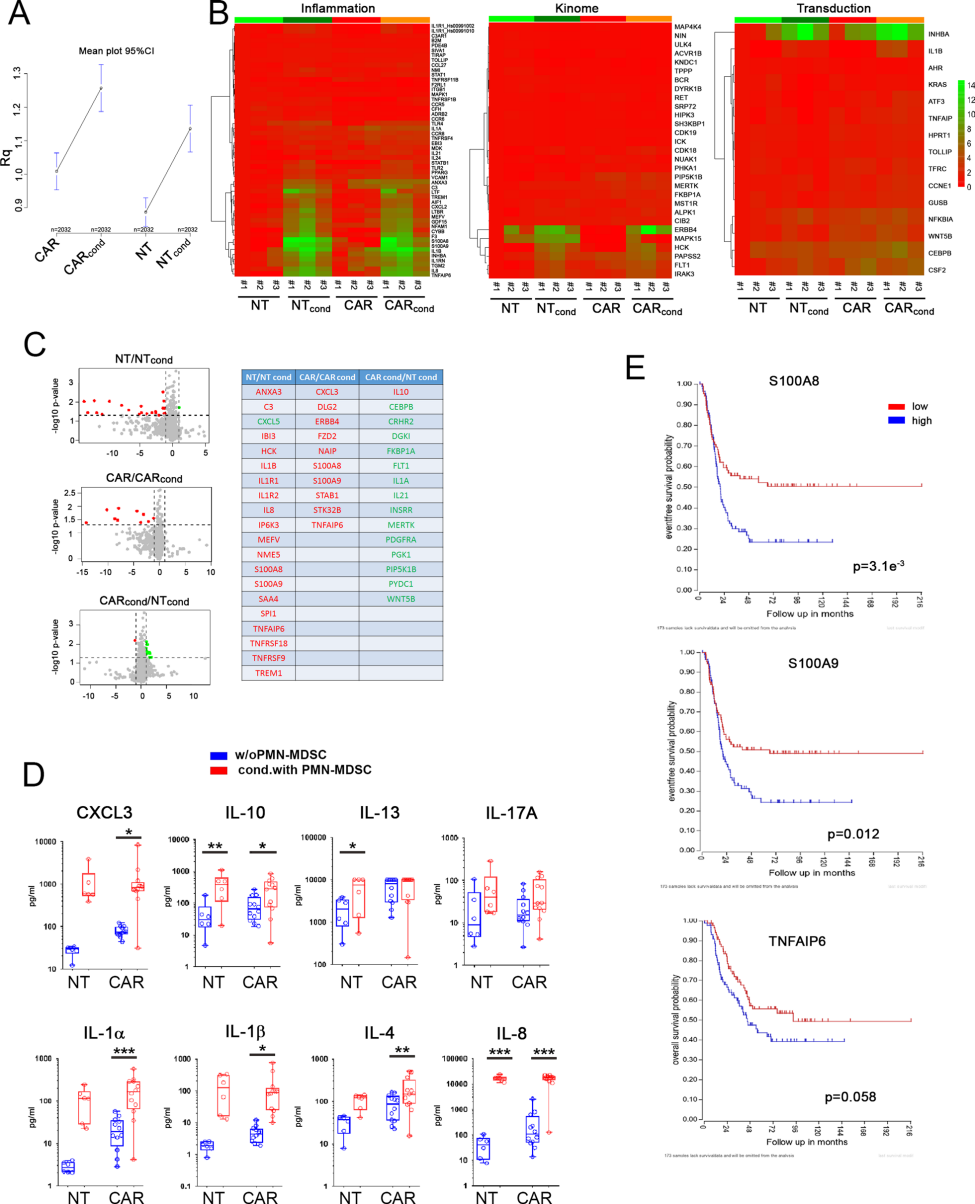
PMN-MDSC induce modulation of the gene-expression profile and inhibit cytokine secretion of GD2.CAR T-cells. **A**–**C** NT and GD2.CAR T-cells were cultured for 48h either in the absence or in the presence (cond.) of PMN-MDSC (NT *n* = 3; GD2.CAR T-cells *n* = 3). **A** Gene expression level (Rq) represented as mean plot with the 95% of confidence interval for the total gene analyzed in NT and GD2.CAR T-cells conditioned or not with PMN-MDSC. **B** Heat-maps representing the unsupervised hierarchical clustering of samples analyzed for inflammation, kinome and signal transduction gene-expression profile. Columns represent samples (NT and GD2.CAR T-cells), rows represent genes. **C** Volcano plots show inflammation, kinome and signal transduction gene expression data. The red or green dots indicate genes-of-interest that display highly positive or negative fold-change (*x* axis) and high statistical significance (− log10 of *p* value, *y* axis). The gene name related to a red and green dot is reported in the table (right panel). **D** Levels (pg/ml) of the indicated cytokines/chemokines in the supernatant of NT (*n* = 6) and GD2.CAR T-cells (*n* = 12) conditioned (red) or not (blue) with PMN-MDSC are shown. **E** Kaplan–Meier curves estimate of overall survival of HR-NB patients according to the level of S100A8 or S100A9 or TNFAIP6 gene expression. Red (low) and blue (high) lines indicate the level of gene expression using median cut-off modus. Dataset used for the analysis is indicated. One-way ANOVA was used for statistical analysis. *p < 0.05, **p < 0.01, ***p < 0.005

S100A8 and S100A9, belonging to the S100 family of proteins containing two canonical EF-hand calcium binding motifs, are involved in the calcium dependent control of cell differentiation, cell cycle progression and growth. Several studies underlined how these two proteins are up-regulated in many cancer patients and demonstrated their role in tumor promotion and progression by inducing MDSC [19] or generating the protein calprotectin, which has been shown to induce T-cell apoptosis and exhaustion [20–22].

The TNFAIP6 is a secretory protein that contains a hyaluronan-binding domain extremely important for T-cell migration within the extracellular matrix. Furthermore, TNFAIP6, like the other family members of TNFAIP, is involved in immune reactions, inflammatory responses, signal transduction, apoptosis, differentiation, material transport and other biological functions. They play important roles in multiple diseases and in the immune and inflammatory processes of cancer, including their overexpression in T-cells [23].

Interestingly, analysis on the up- and down-regulated genes showed upregulation of Th2 cytokines, activation of senescence pathways and sensitivity to TNF signaling, as well as down-regulation of genes involved in pathways controlling the intracellular signaling, metabolic process and inflammasome in conditioned GD2.CAR T-cells as compared to un-conditioned GD2.CAR T-cells (Additional file 5: Fig. S5B–D).

To further validate our data and verify the effect of the S100A8, S100A9 and TNFAIP6, we queried the public R2 NB gene-expression data set and found a strong correlation between upregulation of S100A8, S100A9 and poor clinical outcome in high-risk (HR)-NB patients and a trend for the TNFAIP6 (Fig. 2E). Moreover, the correlation between upregulation of S100A8, S100A9 and poor patient outcome has been confirmed also on the full NB population (Additional file 5: Fig. S5E). As previously shown, PMN-MDSC are able to inhibit different immune effector cells, including NK [9], αβ- and γδ-T-cells [24]. Moreover, inflammatory mediators, such as IL8 and IL1β and IL13, drive their accumulation and contribute to their inhibitory activity. In addition, MDSC can induce macrophage polarization towards a M2 profile, inhibit NK cell-mediated anti-tumor activity and recruit regulatory T-cells [25]. Of note, the different immunophenotypic characteristics of MDSC and their suppressive mechanisms of MDSCs reflect their heterogeneous nature [25, 26]. Therefore, different approaches are required for targeting human MDSC. Several compounds, including ATRA (all-trans-retinoic acid) [27, 28], vitamin D [29] and paclitaxel [30], have been shown to be able to block the MDSC immunosuppressive activity by inducing their differentiation. Notably, chemotherapeutic agents (e.g. gemcitabine or 5-fluorouracil) [31, 32] directly reduce the frequency of MDSC enhancing the anti-tumor immune activity.

Overall, our data demonstrate the need to further implement the design of both clinical trials and CAR constructs and highlights the prognostic relevance of PMN-MDSC, since these cells are able to suppress in general anti-tumor effector cells, and not only CAR T-cells. Therapeutic agents capable of targeting and neutralizing PMN-MDSC might help restoring an effective anti-tumor activity of effector cells and protect CAR T-cells from the detrimental action displayed by immunosuppressive cells.

In conclusion, PMN-MDSC may represent a novel target in tumor immunotherapy and an important biomarker predicting response to immunotherapy-based treatments, not necessarily limited to CAR T-cell-based approaches. Whether these cells may interfere with conventional chemotherapy is an issue to be investigated in future ad hoc designed studies.

## Supplementary Information

**Additional file 1: Fig. S1.** Functional test to assess the PMN-MDSC inhibitory capability. (A-D) T-cells isolated from healthy donor PB or GD2.CAR T-cells were co-cultured either in the absence or in the presence of PMN-MDSC derived from PB of NB patient. The proliferation capability was assessed by flow-cytometry for T-cells (A) or GD2.CAR T-cells (C, n = 6) after 5 or 4 days, respectively. The number of cells for each division is indicated. (B and D) TNF-α and IFN-γ production by CD8^+^ and CD8^−^ T-cells (B) or GD2.CAR T-cells (D, n = 6) upon over-night stimulation with PMA and Ionomycin.

**Additional file 2: Fig. S2.** Schematic representation of the retroviral transduction. (A) Schematic representation of the CAR constructs. Second-generation CAR T-cell constructs encoding CD28 or OX40 or 4-1BB costimulatory molecules. Third-generation CAR T-cell constructs encoding CD28.OX40 or CD28.4-1BB costimulatory molecules. All transduced CAR T-cells were equipped with the signal endo-domain derived from the CD3ζ chain. (B) Retroviral vector codifying for an eGFP. Created with Biorender.

**Additional file 3: Fig. S3.** Effect of PMN-MDSC on third-generation GD2.CAR T-cells. Third-generation GD2.CAR T-cells were cultured either in the absence (w/o PMN-MDSC) or in the presence (with PMN-MDSC, 1:1) of PMN-MDSC collected from stem cell donors given G-CSF for hematopoietic stem cell mobilization and undergoing to leukapheresis. After 48 hours, GD2.CAR T-cells and non-transduced (NT) cells (used as control) were collected, purified and co-cultured at the effector:target ratio 5:1 with the SH-SY5Y-eGFP NB cell line. Percentages of SH-SY5Y-eGFP^+^ NB residual live cells at day 3 of co-culture third-generation GD2.CAR T-cells (n = 7).

**Additional file 4: Fig. S4.** Schematic representation of how PMN-MDSC compromise the GD2.CAR T-cell based therapy. (A) CAR T-cell preparation and infusion. (B) Differentiation steps from common myeloid precursors (pink cells) towards neutrophils (blue cells). Tumor cells (brown cells) and the TME may induce neutrophil accumulation and differentiation towards PMN-MDSC (dark grey cells). (C-D) Upon GD2.CAR T-cell infusion, patients could either (C) respond to CAR treatment (Responders) or (D) could display accumulation of PMN-MDSC which inhibit GD2.CAR T-cell expansion/function, thus contributing to the lack of efficacy of CAR T-cell therapy (Non-Responders). Created with Biorender.

**Additional file 5: Fig. S5.** Expression of informative genes in GD2.CAR T-cells upon interaction with PMN-MDSC. (A) Gene expression level (Rq) represented as mean plot with the 95% of confidence interval in NT and GD2.CAR T-cells conditioned or not with PMN-MDSC for the indicated gene arrays: inflammation (n = 607 genes), kinome (n = 828 genes) and signal transduction (n = 597 genes). (B) Gene analysis correlation between S100A8, S100A9 and TNFAIP6 transcripts and all genes analyzed with p ≤ 0.01. (C) Pathway enrichment analysis for S100A8, S100A9 and TNFAIP6 genes. Clusters of different pathways were visualized in different colors, with the size of rectangles adjusted to reflect their p-value. (D) Pathway enrichment analysis on the up- and down-regulated genes in CAR/CAR conditioned T-cells. (E) Overall survival of NB patients (low, medium and HR) and level of S100A8 or S100A9 or TNFAIP6 gene expression. Red (low) and blue (high) lines indicate the level of gene expression using median cut-off modus. Dataset used for the analysis is indicated.

**Additional file 6.** Supplementary materials and methods.

## Data Availability

All data obtained during the current study are available from the corresponding authors.

## Abbreviations

NB: Neuroblastoma
GD2: Disialoganglioside 2
CAR: Chimeric antigen receptor
PMN-MDSC: Polymorphonuclear myeloid-derived suppressor cells
PB: Peripheral blood
HR: High-risk
EFS: Event-free survival
TME: Tumor microenvironment
MDSC: Myeloid-derived suppressor cells
PMA: Phorbol 12-myristate 13 acetate
Iono: Ionomycin
TNF: Tumor Necrosis Factor
IFN: Interferon
G-CSF: Granulocyte colony-stimulating factor
NT: Non-transduced
eGFP: Enhanced green fluorescence protein
CXCL3: C-X-C Motif Chemokine Ligand 3
BM: Bone Marrow
Tregs: Regulatory T cells
FBS: Fetal bovine serum
DMEM: Dulbecco's Modified Eagle's Medium
scFv: Single chain variable fragment
OPBG: Bambino Gesù Children’s Hospital
S100: S100 calcium binding protein
TNFAIP6: TNF Alpha Induced Protein 6
SEM: Standard error mean
