# Supplementary figures and images for: Polymorphonuclear myeloid-derived suppressor cells impair the anti-tumor efficacy of GD2.CAR T-cells in patients with neuroblastoma
Source: J Hematol Oncol. 2021 Nov 12;14:191. doi: 10.1186/s13045-021-01193-0 (PMC8588686; doi:10.1186/s13045-021-01193-0)

A

Gated on T-cells

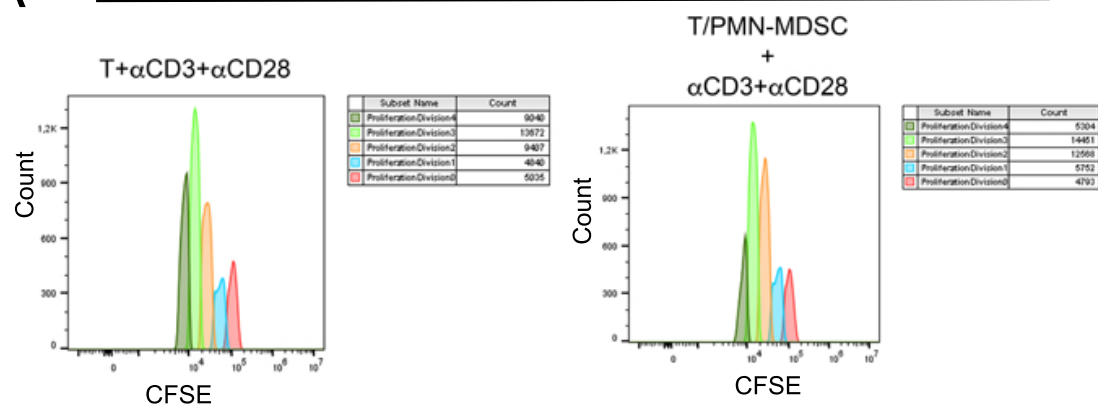

B

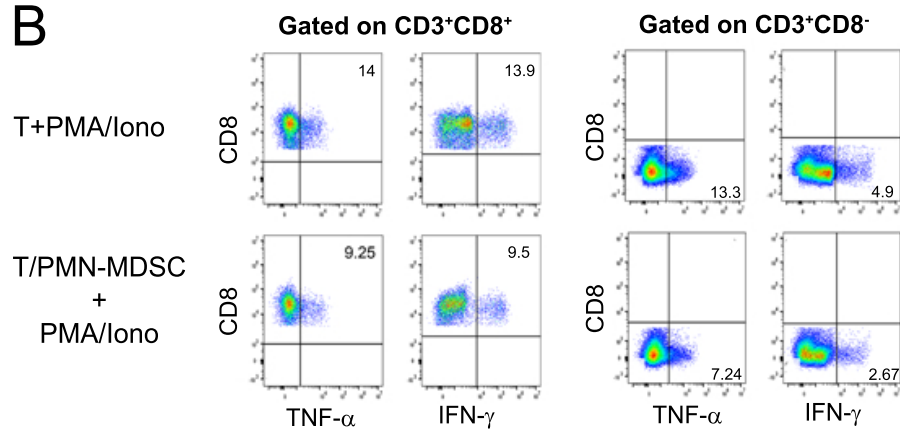

C

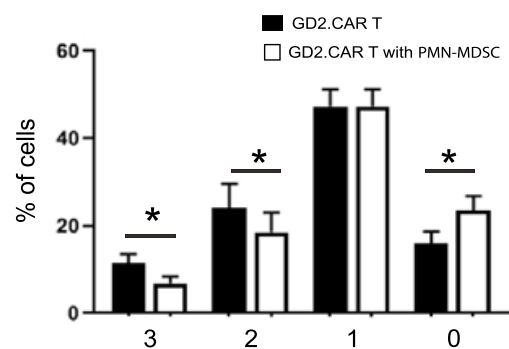

D

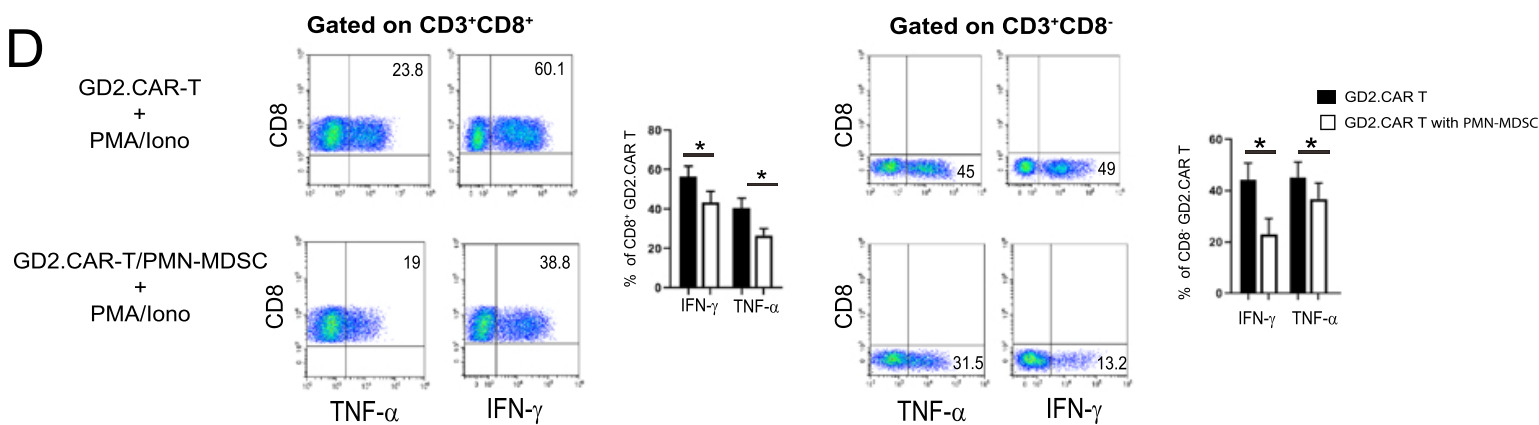

Supplement: Supplementary file 1 — Additional file 1: Fig. S1. Functional test to assess the PMN-MDSC inhibitory capability. (A-D) T-cells isolated from healthy donor PB or GD2.CAR T-cells were co-cultured either in the absence or in the presence of PMN-MDSC derived from PB of NB patient. The proliferation capability was assessed by flow-cytometry for T-cells (A) or GD2.CAR T-cells (C, n = 6) after 5 or 4 days, respectively. The number of cells for each division is indicated. (B and D) TNF-α and IFN-γ production by CD8+ and CD8− T-cells (B) or GD2.CAR T-cells (D, n = 6) upon over-night stimulation with PMA and Ionomycin. [file 13045_2021_1193_MOESM1_ESM.pdf]

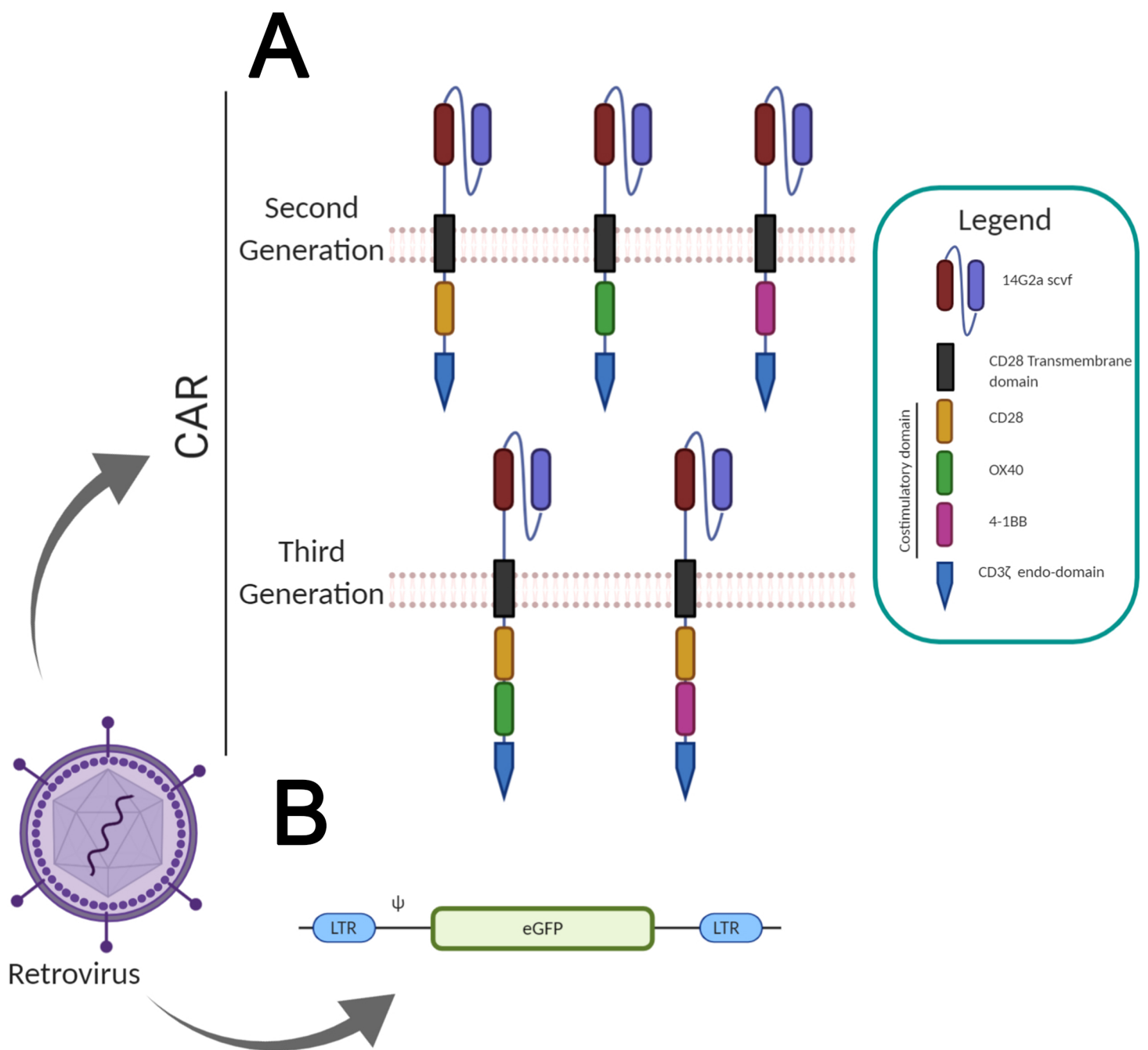

Supplementary Figure 2

Supplement: Supplementary file 2 — Additional file 2: Fig. S2. Schematic representation of the retroviral transduction. (A) Schematic representation of the CAR constructs. Second-generation CAR T-cell constructs encoding CD28 or OX40 or 4-1BB costimulatory molecules. Third-generation CAR T-cell constructs encoding CD28.OX40 or CD28.4-1BB costimulatory molecules. All transduced CAR T-cells were equipped with the signal endo-domain derived from the CD3ζ chain. (B) Retroviral vector codifying for an eGFP. Created with Biorender. [file 13045_2021_1193_MOESM2_ESM.pdf]

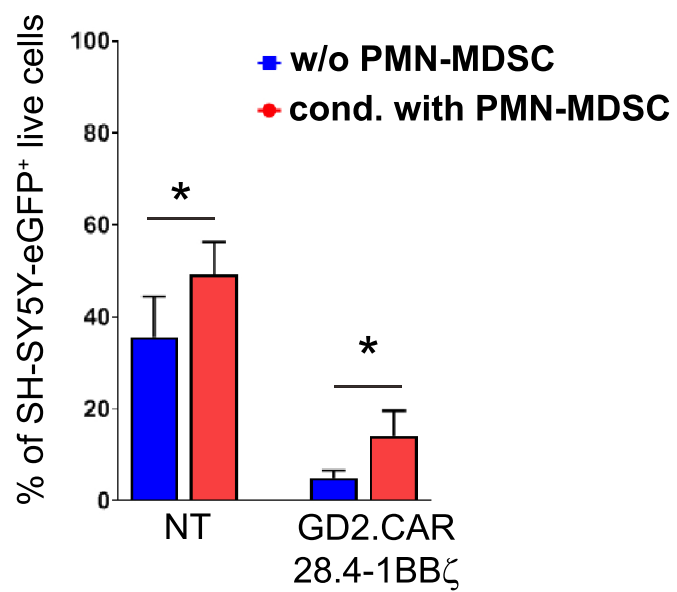

Supplementary Figure 3

Supplement: Supplementary file 3 — Additional file 3: Fig. S3. Effect of PMN-MDSC on third-generation GD2.CAR T-cells. Third-generation GD2.CAR T-cells were cultured either in the absence (w/o PMN-MDSC) or in the presence (with PMN-MDSC, 1:1) of PMN-MDSC collected from stem cell donors given G-CSF for hematopoietic stem cell mobilization and undergoing to leukapheresis. After 48 hours, GD2.CAR T-cells and non-transduced (NT) cells (used as control) were collected, purified and co-cultured at the effector:target ratio 5:1 with the SH-SY5Y-eGFP NB cell line. Percentages of SH-SY5Y-eGFP+ NB residual live cells at day 3 of co-culture third-generation GD2.CAR T-cells (n = 7). [file 13045_2021_1193_MOESM3_ESM.pdf]

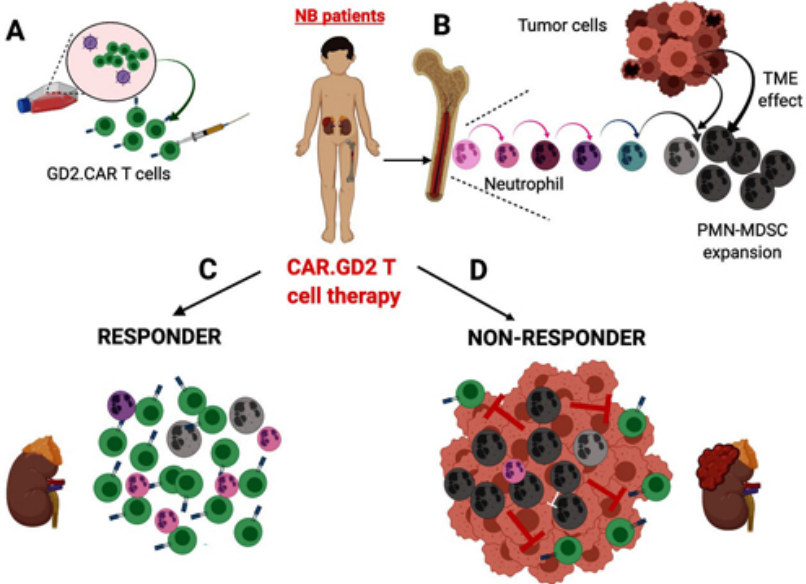

Supplementary Figure 4

Supplement: Supplementary file 4 — Additional file 4: Fig. S4. Schematic representation of how PMN-MDSC compromise the GD2.CAR T-cell based therapy. (A) CAR T-cell preparation and infusion. (B) Differentiation steps from common myeloid precursors (pink cells) towards neutrophils (blue cells). Tumor cells (brown cells) and the TME may induce neutrophil accumulation and differentiation towards PMN-MDSC (dark grey cells). (C-D) Upon GD2.CAR T-cell infusion, patients could either (C) respond to CAR treatment (Responders) or (D) could display accumulation of PMN-MDSC which inhibit GD2.CAR T-cell expansion/function, thus contributing to the lack of efficacy of CAR T-cell therapy (Non-Responders). Created with Biorender. [file 13045_2021_1193_MOESM4_ESM.pdf]

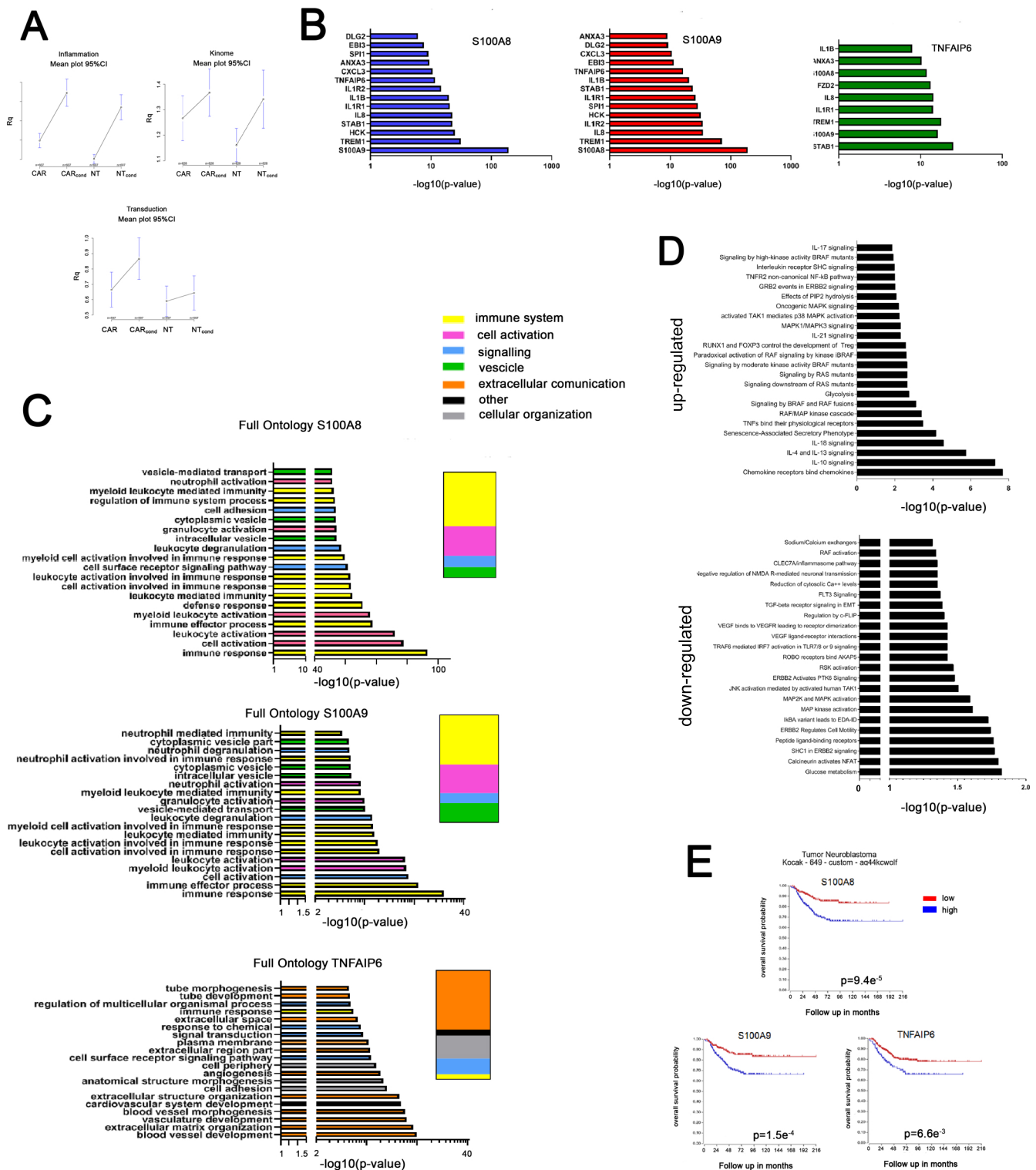

Supplementary Figure 5

Supplement: Supplementary file 5 — Additional file 5: Fig. S5. Expression of informative genes in GD2.CAR T-cells upon interaction with PMN-MDSC. (A) Gene expression level (Rq) represented as mean plot with the 95% of confidence interval in NT and GD2.CAR T-cells conditioned or not with PMN-MDSC for the indicated gene arrays: inflammation (n = 607 genes), kinome (n = 828 genes) and signal transduction (n = 597 genes). (B) Gene analysis correlation between S100A8, S100A9 and TNFAIP6 transcripts and all genes analyzed with p ≤ 0.01. (C) Pathway enrichment analysis for S100A8, S100A9 and TNFAIP6 genes. Clusters of different pathways were visualized in different colors, with the size of rectangles adjusted to reflect their p-value. (D) Pathway enrichment analysis on the up- and down-regulated genes in CAR/CAR conditioned T-cells. (E) Overall survival of NB patients (low, medium and HR) and level of S100A8 or S100A9 or TNFAIP6 gene expression. Red (low) and blue (high) lines indicate the level of gene expression using median cut-off modus. Dataset used for the analysis is indicated. [file 13045_2021_1193_MOESM5_ESM.pdf]
