# Supplementary material for: Polymorphonuclear myeloid-derived suppressor cells impair the anti-tumor efficacy of GD2.CAR T-cells in patients with neuroblastoma
Source: J Hematol Oncol. 2021 Nov 12;14:191. doi: 10.1186/s13045-021-01193-0 (PMC8588686; doi:10.1186/s13045-021-01193-0)
Supplement: Supplementary file 6 — Additional file 6. Supplementary materials and methods. [file 13045_2021_1193_MOESM6_ESM.docx]

**Material and Methods**

**Cell lines** All tumor cell lines were purchased from DSMZ (Braunschweig, Germany). The NB SH-SY5Y was cultured in DMEM (Thermo Scientific, Pittsburgh, PA, USA) supplemented with 10% fetal bovine serum (FBS) and 2 mM Glutamax (Thermo Scientific). The 293T cell line was cultured in IMDM (Thermo Scientific), supplemented with 10% FBS and 2 mM Glutamax. Cells were maintained in a humidified atmosphere containing 5% CO_2_ at 37°C. All cell lines were routinely tested for mycoplasma infection and authenticated by short tandem repeat analysis (Eurofins Genomic, Ebersberg, Germany). PMN-MDSC were isolated by CD66b microbeads kit (Miltenyi, Bergisch Gladbach, Germany), following manufacturer’s instructions (purity>98%) from G-CSF mobilized adult healthy donors that undergo mobilization for hematopoietic stem cell transplantation and NB patients enrolled in the phase I/II clinical trial (NCT03373097). Cell viability was assessed by flow-cytometry analysis (Propidium Iodide, Sigma Aldrich, Saint Louis, USA).

**Retroviral constructs, transient transfection and transduction of T lymphocytes** Using the GD2-specific antibody single chain variable fragment (scFv) (14.G2a), we generated second- and third-generation CAR encoding the common costimulatory molecules CD28, OX40 and 4-1BB and the signal endo-domain derived from the CD3ζ chain (Supplementary Fig. S1)(1). An additional retroviral vector encoding eGFP was used to label the SH-SY5Y tumor cells. Transient retroviral supernatant was produced as previously described(2). T lymphocytes were activated from PB mononuclear cells derived from buffy coats obtained from healthy donors at Bambino Gesù Children’s Hospital (OPBG) after informed consent was signed, in accordance with the rules set by our Institutional Review Board (OPBG - Ethical approval committee, Rome, with prot. N°969/2015) with immobilized OKT3 (1 μg/ml, e-Bioscience; San Diego, CA, USA) and anti-CD28 (1 μg/ml, BD Biosciences, San Jose, CA, USA) mAb in complete medium consisting of 45% RPMI1640, 45% Click’s medium (Sigma-Aldrich) and supplemented with 10% FBS and 2 mM Glutamax, in a humidified atmosphere containing 5% CO_2_ at 37°C and recombinant human IL7/15 (10ng/ml and 5ng/ml, respectively, R&D, Minneapolis, MN, USA). Then, T-cells were transduced with retroviral supernatants using retronectin-coated non-tissue-culture treated 24-well plates (Takara Bio, Shiga, Japan). The transduction efficiency was determined by anti-CAR idiotype staining (1A7), as previously described (3).

**Patients** In this study, we analyzed samples derived from pediatric patients (age 2-18 years n=16), affected by refractory or relapsed NB, enrolled in a phase I/II clinical trial with third-generation CAR T-cells redirected towards the GD2 antigen, through a construct that includes CD28 and 4-1BB as costimulatory molecules, at IRCCS Bambino Gesù Children’s Hospital, in Rome (Italy) (NCT03373097). All patients or legal guardians provided written informed consent and the whole research was conducted under institutional review board approved protocols, in accordance with the Declaration of Helsinki.

**Phenotypic analysis** Expression of cell surface molecules was determined by flow-cytometry using standard methodology after peripheral blood mononuclear cell separation by density gradient centrifugation as reported before(1, 4). The following mAbs were used: CD3, CD4, CD8, CD11b, CD14, CD15, CD16, CD19, CD25, CD33, CD45, CD56, CD66b, GD2, and HLA-DR. Duraclone platform (Beckman Coulter, Brea, CA, USA) was used for MDSC identification. These mAb were purchased by BD Bioscience, Miltenyi, BioLegend (San Diego, CA, USA) and Beckman Coulter. Samples were analyzed on a BD LSRFortessa X-20 (BD Biosciences), Cytoflex S and LX (Beckman Coulter). Data were analyzed using Cytexpert (Beckman Coulter) and FlowJo 10 software (BD Biosciences). For each sample, we analyzed a minimum of 50,000 events.

**Functional assays** For co-culture experiments, T-cells isolated from healthy donor PB or NT and GD2.CAR T-cells generated from healthy donors were co-cultured in the presence or absence of allogeneic PMN-MDSC (derived from PB of NB patients or from stem cell donors given G-CSF and undergoing to leukapheresis) at 1:1 ratio. Subsequently, T cell proliferation, cytokine production and cytotoxicity were assessed. For cell proliferation, T lymphocytes were labelled with CFSE (Invitrogen) following manufacturing instructions and stimulated with anti-CD3 (1ug/ml, Miltenyi) and anti-CD28 (5ug/ml, Miltenyi). For cytokine production, cells were stimulated with PMA (25ng/ml) and Ionomycin (1ug/ml) + Brefeldin A (Golgi Plug, BD) for 18 hours and analyzed as previously described(4). For cytotoxicity, upon co-culture with PMN-MDSC, GD2.CAR T-cells were isolated and further co-cultured at 5:1 or 1:1 E:T ratio with the SHSY5Y-eGFP^+^ cell line. Following 3 or 5 days of incubation at 37°C, residual tumor cells and T-cells were assessed by flow-cytometry analysis based on CD3 (Effector T-cells) and eGFP (NB tumor) expression.

**Cytokine profile** Supernatant from co-cultures was collected at 24 hours after co-culture to measure cytokine release. Cytokines were measured by MILLIPLEX MAP Human Magnetic Bead Panel assay (Merck, Darmstadt - Germany), using a MAGPIX^®^ with xPONENT^®^ software, following the manufacturer’s instructions. In particular, we investigated the following cytokines: IL1α, IL1β, IL4, IL8, IL10, IL13, IL17a and CXCL3.

**Gene expression** RNA of NT and GD2.CAR T-cells, conditioned or not with PMN-MDSC, was isolated using the RNeasy Mini Kit (Qiagen, Hilden, Germany) and reversely transcribed with the cDNA SuperScript™ VILO™ cDNA Synthesis Kit (Thermo Scientific). Samples were pre-amplified following the manufacturer’s instructions. Gene expression was then tested using the Human Signal Transduction, Inflammation and Kinoma TaqMan® OpenArray® Pathways Panels (Thermo Scientific). A 12K qPCR QuantStudio Flex Real-Time PCR system was used to perform the run as previously described (5).

**Statistical analysis** Unless otherwise noted, data are summarized as mean ± SEM. Student t-test (two-sided) was used to determine statistically significant differences between samples, p-value <0.05 indicating a significant difference. When multiple comparison analyses were required, statistical significance was evaluated by repeated measures ANOVA followed by a Log-rank (Mantel-Cox) test. The overall survival data according to S100A8, S100A9 and TNFAIP6 expression were analyzed using R2: Genomics Analysis and Visualization Platform (http://r2.amc.nl) with the selection of a median cut-off modus. Pathway enrichment analysis was performed with “Reactome pathway data set” (www.reactome.org) on the set of differentiated expressed genes. No valuable samples were excluded from the analyses. Graph generation and statistical analyses were performed using Prism version 8 software (GraphPad, La Jolla, CA - USA).

**Consent for publication**

All the authors reviewed the manuscriptAQ4 and consented to its submission and further publication in Journal Hematology and Oncology.

**Supplementary Figure 1. Functional test to assess the PMN-MDSC inhibitory capability.** (A-B) T-cells isolated from healthy donor PB or GD2.CAR T-cells were co-cultured either in the absence or in the presence of PMN-MDSC derived from PB of NB patient. The proliferation capability was assessed by flow-cytometry for T-cells (A) or GD2.CAR T-cells (C, n=6) after 5 or 4 days, respectively. The number of cells for each division is indicated. (B and D) TNF-α and IFN-γ production by CD8^+^ and CD8^-^ T-cells (B) or GD2.CAR T-cells (D, n=6) upon over-night stimulation with PMA and Ionomycin.

**Supplementary Figure 2. Schematic representation of the retroviral transduction**. (A) Schematic representation of the CAR constructs. Second-generation CAR T-cell constructs encoding CD28 or OX40 or 4-1BB costimulatory molecules. Third-generation CAR T-cell constructs encoding CD28.OX40 or CD28.4-1BB costimulatory molecules. All transduced CAR T-cells were equipped with the signal endo-domain derived from the CD3ζ chain. (B) Retroviral vector codifying for an eGFP. Created with Biorender.

**Supplementary Figure 3. Effect of PMN-MDSC on third-generation GD2.CAR T-cells.**

Third-generation GD2.CAR T-cells were cultured either in the absence (w/o PMN-MDSC) or in the presence (with PMN-MDSC, 1:1) of PMN-MDSC collected from stem cell donors given G-CSF for hematopoietic stem cell mobilization and undergoing to leukapheresis. (A) After 48 hours, GD2.CAR T-cells and non-transduced (NT) cells (used as control) were collected, purified and co-cultured at the effector:target ratio 5:1 with the SH-SY5Y-eGFP NB cell line. Percentages of SH-SY5Y-eGFP^+^ NB residual live cells at day 3 of co-culture third-generation GD2.CAR T-cells (n=7).

**Supplementary Figure 4. Schematic representation of how PMN-MDSC compromise the GD2.CAR T-cell based therapy.** (A) CAR T-cell preparation and infusion. (B) Differentiation steps from common myeloid precursors (pink cells) towards neutrophils (blue cells). Tumor cells (brown cells) and the TME may induce neutrophil accumulation and differentiation towards PMN-MDSC (dark grey cells). (C-D) Upon GD2.CAR T-cell infusion, patients could either (C) respond to CAR treatment (Responders) or (D) could display accumulation of PMN-MDSC which inhibit GD2.CAR T-cell expansion/function, thus contributing to the lack of efficacy of CAR T-cell therapy (Non-Responders). Created with Biorender.

**Supplementary Figure 5. Expression of informative genes in GD2.CAR T-cells upon interaction with PMN-MDSC.** (A) Gene expression level (Rq) represented as mean plot with the 95% of confidence interval in NT and GD2.CAR T-cells conditioned or not with PMN-MDSC for the indicated gene arrays: inflammation (n=607 genes), kinome (n=828 genes) and signal transduction (n=597 genes). (B) Gene analysis correlation between S100A8, S100A9 and TNFAIP6 transcripts and all genes analyzed with p≤0.01. (C) Pathway enrichment analysis for S100A8, S100A9 and TNFAIP6 genes. Clusters of different pathways were visualized in different colors, with the size of rectangles adjusted to reflect their p-value. (D) Pathway enrichment analysis on the up- and down-regulated genes in CAR/CAR conditioned T-cells. (E) Overall survival of NB patients (low, medium and HR) and level of S100A8 or S100A9 or TNFAIP6 gene expression. Red (low) and blue (high) lines indicate the level of gene expression using median cut-off modus. Dataset used for the analysis is indicated.

**Supplementary references**

1. Quintarelli C, Orlando D, Boffa I, Guercio M, Polito VA, Petretto A, et al. Choice of costimulatory domains and of cytokines determines CAR T-cell activity in neuroblastoma. Oncoimmunology. 2018;7(6):e1433518.

2. Caruana I, Savoldo B, Hoyos V, Weber G, Liu H, Kim ES, et al. Heparanase promotes tumor infiltration and antitumor activity of CAR-redirected T lymphocytes. Nature medicine. 2015;21(5):524-9.

3. Caruana I, Weber G, Ballard BC, Wood MS, Savoldo B, Dotti G. K562-Derived Whole-Cell Vaccine Enhances Antitumor Responses of CAR-Redirected Virus-Specific Cytotoxic T Lymphocytes In Vivo. Clin Cancer Res. 2015;21(13):2952-62.

4. Tumino N, Besi F, Di Pace AL, Mariotti FR, Merli P, Li Pira G, et al. PMN-MDSC are a new target to rescue graft-versus-leukemia activity of NK cells in haplo-HSC transplantation. Leukemia. 2020;34(3):932-7.

5. Polito VA, Cristantielli R, Weber G, Del Bufalo F, Belardinilli T, Arnone CM, et al. Universal Ready-to-Use Immunotherapeutic Approach for the Treatment of Cancer: Expanded and Activated Polyclonal gammadelta Memory T Cells. Front Immunol. 2019;10:2717.
